# Monitoring and evaluation of malaria in pregnancy – developing a rational basis for control

**DOI:** 10.1186/1475-2875-7-S1-S6

**Published:** 2008-12-11

**Authors:** Bernard J Brabin, Marian Wasame, Ulrika Uddenfeldt-Wort, Stephanie Dellicour, Jenny Hill, Sabine Gies

**Affiliations:** 1 Child and Reproductive Health Group, Liverpool School of Tropical Medicine, Liverpool L3 5QA, UK; 2 Global Child Health Group, Emma Kinderziekenhuis, Academic Medical Centre, University of Amsterdam, The Netherlands; 3 Global Malaria Programme, World Health Organization, 20 Avenue Appia, Geneva, Switzerland; 4 Social Medicine and Global Child Health, Clinical Research Sciences, Lund University, Lund, Sweden; 5 Epidemiology and Control of Parasitic Diseases Unit, Department of Parasitology, Prince Leopold Institute of Tropical Medicine, Antwerp, Belgium

## Abstract

Monitoring and evaluation of malaria control in pregnancy is essential for assessing the efficacy and effectiveness of health interventions aimed at reducing the major burden of this disease on women living in endemic areas. Yet there is no currently integrated strategic approach on how this should be achieved. Malaria control in pregnancy is formulated in relation to epidemiological patterns of exposure. Current emphasis is on intermittent preventive treatment (IPTp) during pregnancy with sulphadoxine-pyrimethamine in higher transmission areas, combined with insecticide treated bed nets (ITNs) and case management. Emphasis in lower transmission areas is primarily on case management. This paper discusses a rational basis for monitoring and evaluation based on: assessments of therapeutic and prophylactic drug efficacy; proportional reductions in parasite prevalence; seasonal effects; rapid assessment methodologies; birthweight and/or anaemia nomograms; case-coverage methods; maternal mortality indices; operational and programmatic indicators; and safety and pharmacovigilance of antimalarials in pregnancy. These approaches should be incorporated more effectively within National Programmes in order to facilitate surveillance and improve identification of high-risk women. Systems for utilizing routinely collected data should be strengthened, with greater attention to safety and pharmacovigilance with the advent of artemisinin combination therapies, and prospects of inadvertent exposures to artemisinins in the first trimester. Integrating monitoring activities within malaria control, reproductive health and adolescent-friendly services will be critical for implementation. Large-scale operational research is required to further evaluate the validity of currently proposed indicators, and in order to clarify the breadth and scale of implementation to be deployed.

## Background

Malaria in pregnancy is an immense public health problem with at least 50 million pregnant women living in malaria endemic areas [1]. Under conditions of high transmission there is increased *Plasmodium falciparum *parasite prevalence in placental and peripheral blood, which is higher in first, compared to later, pregnancies. This epidemiological pattern for *P. falciparum *has been recognized for many years, with higher prevalence in early pregnancy which increases, if untreated, and then decreases as gestation progresses [2-4]. In stable high transmission areas, almost all primigravidae, if unprotected, are likely to be infected in early pregnancy and approximately half of these would remain infected by the time of delivery if untreated. In multigravidae, especially in higher parities, prevalence is significantly reduced due to the acquisition of parity-specific immunity [5]. Maternal HIV infection is associated with increased *P. falciparum *prevalence and delays clearance of parasitaemia in multigravidae.

**Figure 1:**
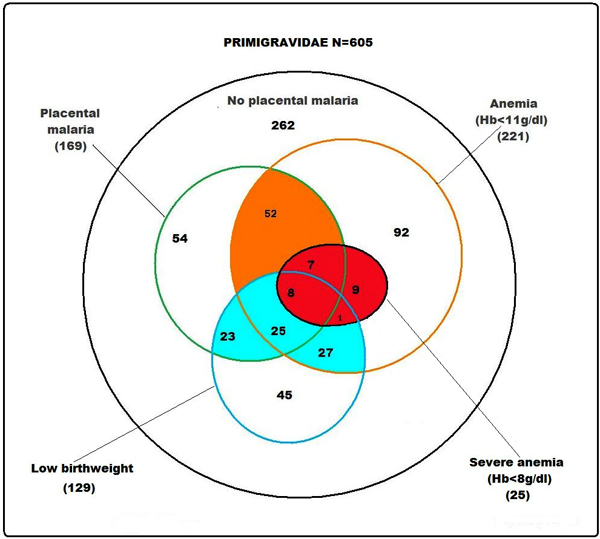
Venn diagram for malaria related pregnancy outcomes in 605 primigravidae. Data source: DELIMAL study, Burkina Faso (S. Gies *et al*, unpublished data and [34]).

Maternal anaemia and low birthweight are two of the important consequences of malaria in pregnancy [6], and Figure 1 illustrates the magnitude and importance of these co-morbidities in primigravidae experiencing *P. falciparum *infection under stable endemic conditions. For low, unstable transmission areas there are fewer studies, but prevalence is greatly reduced (mostly <10%), fewer primigravidae are exposed, and as a result there is reduced development of parity specific immunity [6]. The longitudinal pattern of *Plasmodium vivax *infection is less well described, but is reported as more common in primigravidae than in multigravidae in low transmission areas, and as associated with poorer birth outcomes, such as reduced birthweight and stillbirths [7,8].

**Figure 2:**
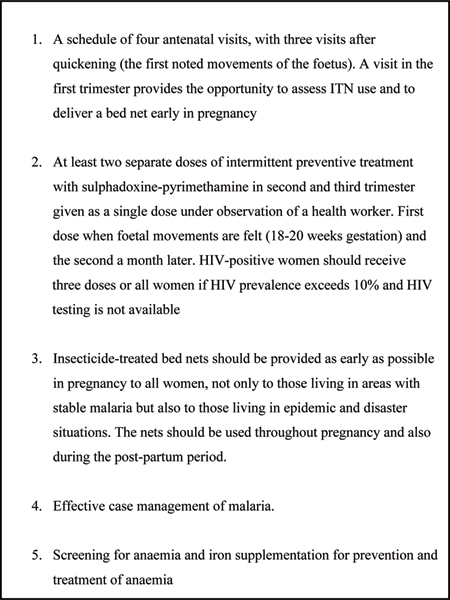
Recommended interventions for malaria control during pregnancy for women living in areas with stable transmission. Source: World Health Organization [1].

Control strategies for malaria in pregnancy are formulated in relation to these epidemiological patterns of infection, with current emphasis in stable transmission areas on intermittent preventive treatment (IPTp) during pregnancy combined with the use of insecticide-treated mosquito nets (ITNs) and case management while, in low transmission areas, emphasis is primarily on case management [1]. Increasing IPTp dosing from two to more frequent doses may be beneficial in some circumstances, but in view of increasing parasite resistance to sulphadoxine-pyrimethamine (SP), the currently recommended antimalarial for IPTp, appropriate monitoring, evaluation and research is required in order to establish optimal control strategies [9]. ITNs used throughout pregnancy, or from mid-pregnancy onwards, improve pregnancy outcomes particularly in the first few pregnancies in women living under malaria endemic conditions in Africa and are recommended by WHO [10]. Their use is a pivotal control option, although further research on their potential benefits in women living in areas outside Africa and under conditions of lower transmission is required. Current recommendations for malaria control during pregnancy in women living in areas with stable transmission are outlined in Figure 2[1].

**Figure 3:**
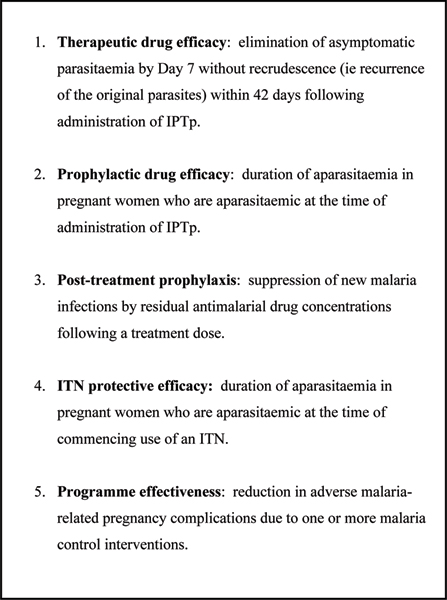
Definition of parameters for assessment of malaria control in pregnancy.

Definitions of parameters related to efficacy and effectiveness of current interventions for malaria control in pregnancy are outlined in Figure 3. These relate to the assessment approaches which can be used and which are discussed below.

### A changing situation

The current global Roll Back Malaria target is that by 2010, 80% of all pregnant women in areas of high stable transmission should receive IPTp. Although most countries in sub-Saharan Africa have adopted this policy, only a small number have achieved widespread programme implementation [11,12]. Although parasite resistance to SP continues to increase it remains an effective strategy for IPTp. Based on the systematic review by ter Kuile *et al*, SP-ITPp was still effective at SP resistance levels from 19%–26%, based on *in vivo *assessments in young children [13]. In view of the varied effectiveness of health systems throughout Africa, these interventions in pregnancy must be balanced against the need for effective monitoring and surveillance programmes assessing progress with implementation (outputs), coverage (outcomes) and protective efficacy (impact). An outline summary of currently recommended indicators for monitoring and evaluation of programmes to control malaria in pregnancy is shown in Table 1. Despite the availability of these guidelines monitoring and evaluation programmes for malaria control in pregnancy are poorly developed. Improved and extended surveillance methods are needed as intervention programmes are more widely adopted and new antimalarial drug options, including artemisinin-based combinations, are introduced. With prompt feedback to programme managers this should serve as a catalyst to improve delivery of interventions for malaria control in pregnancy. Monitoring trends and continued evaluation will allow prevention recommendations to evolve as the risk of infection in women and effectiveness of antimalarial drugs change. Demonstration of the burden of malaria during pregnancy can then be used for directing national malaria programme efforts. A further issue is that as effective strategies are rolled out, and transmission falls, countries with previously high transmission will need to adapt their control strategies, for example, by reducing IPTp and focusing on case detection, diagnosis and treatment.

**Table 1:** Summary of WHO recommended indicators for monitoring and evaluation of programmes to control malaria during pregnancy.

**Output indicators**
1. Percentage of antenatal clinic staff (pre-service, in-service or at supervisory visits) trained in control of malaria during pregnancy in the past 12 months (including IPT, counselling on ITN use and case management for pregnancy women).
2. Percentage of health facilities reporting stock-out of the recommended drug for IPT (currently sulphadoxine-pyrimethamine) in the past month.

**Outcome indicators**

3. Percentage of pregnant women attending antenatal care who receive IPT under direct observation (first dose, second dose, third dose).
4. Percentage of pregnant women who report having slept under an ITN the previous night.

**Impact indicators**

5. Percentage of low birth-weight singleton live births (< 2500 g), by parity.
6. Percentage of screened pregnant women with severe anaemia (haemoglobin 7 g/dl) in third trimester 7, by gravidity.

Source: World Health Organization [14].

To assess progress in the delivery of interventions for the control of malaria in pregnancy, and their effectiveness, a number of core indicators of process, outcome and impact have been identified in order to guide national programmes [14]. In this paper, some new indicators, are reviewed as well as established assessment approaches, for monitoring and evaluation of malaria control in pregnancy and the rational basis for their implementation.

### Antimalarial drug efficacy

The Global Malaria Programme of the World Health Organization (WHO) is developing a framework to monitor and evaluate the efficacy and effectiveness of SP-ITPp on pregnancy outcome in the context of SP resistance and in the absence of continued SP testing in children which previously has been used as a proxy for assessing resistance. The mechanism of action of SP-based IPTp is not yet well established, despite the widely advocated use of the strategy in high transmission settings. SP, a long half-life drug, is expected to be effective by both clearing malaria infections and through post-treatment prophylaxis. For monitoring SP efficacy for IPTp, both therapeutic and preventive efficacy of the medicine, therefore, needs to be assessed.

There is a well-established standard methodology for monitoring antimalarial therapeutic efficacy, which involves repeated assessment of clinical and parasitological responses to treatment during a fixed period of follow-up in order to detect any reappearance of symptoms/signs and/or parasites in the blood as an indication of reduced sensitivity to a particular drug. There is currently no standard protocol for monitoring preventive efficacy, an important component of IPT. Evidence suggests that re-infections of falciparum malaria occur progressively earlier as resistance intensifies, shortening the post-treatment prophylaxis period [15]. Therefore, both the rate of re-infection and duration of time-to-reinfection could be useful indicators for measuring the preventive efficacy of IPTp. These measurements should be related to the desired outcomes of IPTp.

**Figure 4:**
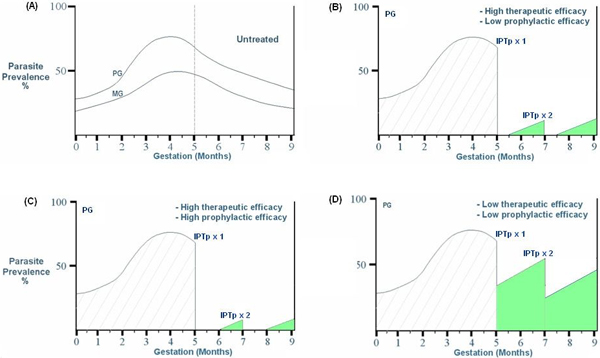
Influence of high and low therapeutic or prophylactic efficacy on parasite burden in primigravidae in high transmission settings. **(A) **Parasite prevalence in primigravidae (PG) and multigravidae (MG) in unprotected pregnancies (from [2]). The fall in parasite prevalence after four months gestation corresponds to the decline observed if untreated and is related to the development of parity-specific immunity; **(B) **High therapeutic (100%) and low prophylactic efficacy (≤50%) with two dose SP-IPTp intermittent preventive treatment; **(C) **High therapeutic (100%) and high prophylactic efficacy (100%); **(D) **Low therapeutic (≤50%) and low prophylactic efficacy (≤50%).

Figure 4 illustrates diagrammatically the reductions using IPTp in proportional parasite burdens during pregnancy in primigravidae in a transmission setting with an entomological inoculation rate (EIR) of about 250 infective bites/year. In these diagrams prevalence represents peripheral parasitaemia. The reductions are estimated for settings with high or low therapeutic and prophylactic SP efficacy. For multigravidae, the parasite burdens would be proportionally lower. With higher EIRs, e.g. 1,000 infective bites/year, then the cumulative incidence would be up to four-fold higher. The patterns of re-infection in these figures are estimated based on a half life of SP in pregnancy of about seven days [16], which is shorter than in non-pregnant women and using a two dose IPTp strategy. Use of drugs with a shorter or longer half-life would increase or decrease, respectively, the areas under the re-infection curves. The sum of the areas under the prevalence curves indicates the overall burden of infection. This illustrates the importance of the first half of pregnancy, which is a period when current antimalarial interventions have limited impact. This period is also not influenced by uptake of ITNs at the first antenatal visit, which is often after 20 weeks gestation. Poor control early in gestation increases foetal loss and the risk of pregnancy anaemia.

Assessing the therapeutic efficacy response to the IPTp first dose (IPTp × 1), can be achieved using the current standard WHO *in vivo *protocol [17], with the following modifications: (i) pregnant women with asymptomatic falciparum infections should be recruited as the study group, (ii) clearance of the parasitaemias at a first antenatal visit in the second trimester, should be monitored, (iii) a 42-day follow-up design should be used, with scheduled visits on days 0, 3, 7, 14, 21, 28 and 42 following administration of IPTp first treatment, (iv) the second IPTp dose would be given following completion of the 42-day follow up, unless rescue treatment was required. According to the WHO *in vivo *protocol [17], the duration of follow-up for therapeutic efficacy is 28 days, and, as the recommended interval between two IPTp doses is at least one month, a 28-day follow-up is practical and logical. The prophylactic effect of SP in infants has been shown to be about 4–5 weeks and one might expect this to be longer in pregnant women infected with parasites of the same level of sensitivity because of the presence of some immunity. For this reason, a 42-day follow-up should be included.

Recrudescence should be differentiated from re-infection through determination of *msp1 *and *msp2 *genotypes using PCR. SP resistance is associated with mutations in the dihydrofolate reductase (*pfdhfr*) and dihydropteroate synthase (*pfdhps*) genes. Characterization of these resistance molecular markers would provide additional information on SP resistance and should be assessed.

Post-treatment prophylaxis could be measured using the above approach assessing the occurrence of new falciparum infections and time to re-infection in uninfected pregnant women within 42 days following administration of IPTp. This could be measured either in previously untreated, aparasitaemic pregnant women, or in women who were parasitaemic and had cleared the parasites after the first SP dose. Either of these options would be suitable, but assessment should not include a combination of both. The proportional reduction in parasite prevalence between the time when IPTp × 1 is received until delivery is an assessment of both therapeutic efficacy and post-treatment prophylaxis. With more frequent IPTp doses this indicator provides an estimate of the total post-treatment prophylaxis effect. This would be a useful composite indicator of both drug resistance and prophylactic efficacy in women receiving IPTp.

### Seasonal effects

What are the monitoring and evaluation implications of malaria seasonality?

**Figure 5:**
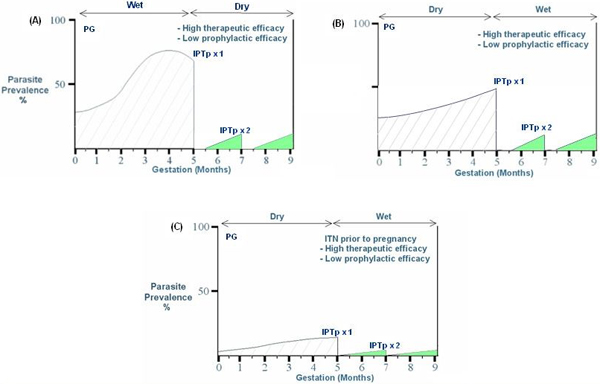
Influence of seasonal effects and ITN use prior to pregnancy on parasite burden in primigravidae (PG). **(A) **First trimester occurring during the wet season and two dose SP-ITPp prescribed in dry season; **(B) **First trimester in the dry season and two dose SP-ITPp prescribed in wet season; **(C) **Good ITN adherence prior to and during first trimester in dry season with subsequent two dose SP-ITPp in wet season. The Entomological Inoculation Rate (EIR) is assumed to be approximately 20/month in the wet season and <5/month in the dry season.

Low EIRs during the dry season do not necessarily equate to low placental parasitaemia. Estimates of dry season prevalence are only slightly lower than those in the wet season [18]. Figure 5 illustrates the influence of season and SP-IPTp efficacy on parasite prevalence, assuming prevalence is marginally lower during the dry season. In the examples shown, ITN use from the time of first antenatal visit (e.g. from five months) would offer small additional protection, which would be greater in the wet season. Figure 5c illustrates that ITN use prior to pregnancy would significantly reduce peripheral parasite prevalence during the first five months of pregnancy, critically reducing the overall pregnancy parasite burden. This emphasizes the importance of assessing net use by, and availability to non-pregnant women as an indicator of adherence prior to pregnancy. It also suggests that strategies proposing use of IPTp in the wet season alone would be a disadvantage to primigravidae experiencing their first trimester in the wet season. These women would not receive IPTp in the wet season, as they are first trimester pregnancies, and then, despite a significant parasite burden, would not receive IPTp during the dry season with a seasonal implementation strategy. Protection with ITNs prior to pregnancy would be required to reduce their risk of first trimester infections. The addition of a new indicator for ITN coverage and use in non-pregnant adolescents is of particular importance in view of any impending first pregnancy.

### Programme effectiveness

#### Rapid assessment methodology

A methodology for this has been published based on cross-sectional prevalence assessments of maternal parasitaemia, maternal anaemia (Hb <11.0 g/dl for moderate anaemia and <7.0 g/dl for severe anaemia) and low birthweight (<2,500 gms), categorized by gravida class [19]. These indicators significantly improve with use of SP-IPTp and/or ITNs [20,21]. Sentinel surveys monitoring these parameters are important for baseline surveys as well as for evaluating subsequent interventions. A slightly higher haemoglobin cut-off, indicating moderately severe anaemia (Hb < 8.0 g/dl), has been frequently used in prevalence assessments and has the advantage that it may be more sensitive to malaria-related changes in haemoglobin concentration in pregnant women living under stable transmission conditions [22].

### Birthweight and anaemia nomograms

**Figure 6:**
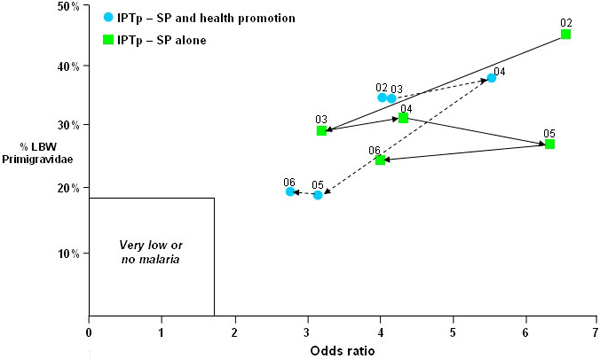
Annual birthweight indicators for singleton pregnancies reported by DELIMAL health centres in Burkina Faso between 2002–2006 (pre-intervention, and intervention April 2004–2006). Point values indicate the year of data collection. The odds ratio refers to excess low birthweight (<2500 g) prevalence in primigravidae compared to multigravidae. Areas receiving SP-ITPp intervention alone (■); areas receiving SP-ITPp combined with a pregnancy health promotion campaign (●).

Approaches for measuring programme effectiveness using routinely collected data should also be considered. Two methodologies have been assessed, based on nomograms for anaemia [22] or birthweight [23], which utilize routinely collected data. Evaluation of birthweight indicators has shown their utility for monitoring malaria exposure and control [24,25], and as birthweight is routinely measured in most delivery facilities, standardization of measurement should allow effective use of nomograms for monitoring. Figure 6 illustrates the use of the birthweight nomogram for evaluation of outcomes of several hundred singleton pregnancies for women delivering in health centres in areas randomized to receive either IPTp-SP alone, or IPT-SP combined with an extended SP health promotion campaign. These data were made available through the DELIMAL study conducted in Burkina Faso between 2004 and 2006 (S.Gies, unpublished data). SP-IPTp was not introduced until April 2004, so that 2002–2003 and part of 2004 were collected under the previous weekly chloroquine prophylaxis policy. In both study arms, birthweight nomograms improve with a directional flow towards reduced low birthweight prevalence (y axis) and lower odds ratios (x axis) with increasing duration of both interventions. There is fluctuation in the indicators between years and the incremental changes are not sequential, although the general direction of change with time is towards improved birth outcomes. Review of these indicators over 3–5 year intervals should demonstrate changing trends.

Maternal anaemia has also been proposed as an indicator for monitoring malaria control in sub-Saharan Africa [26], based on the excess of risk of anaemia in primigravidae compared to multigravidae. This difference is usually observed at first antenatal clinic attendance in women living in malarious areas. An anaemia nomogram has been developed to assess changes in this excess risk with altered malaria exposure [26]. This requires systematic evaluation in view of its combined utility for use in antenatal clinics as well as for monitoring malaria control in pregnancy. It provides a standardized assessment format for comparative evaluations. Sensitivity and specificity estimates are good using either moderate (Hb <11.0 g/dl), or severe anaemia (Hb <8.0 g/dl), for the cut-off values [26].

A key issue is the quality and frequency of assessment of antenatal haemoglobin in most countries in sub-Saharan Africa. This is becoming an increasingly important issue in view of the sustained, high prevalence of anaemia, often severe, in women living in malarious areas and which is not attributable to iron deficiency alone [22,27]. Screening, recognition and appropriate management of pregnancy anaemia is a pivotal aspect of antenatal care and strategies of assessment should be more effectively linked to the monitoring of malaria control in pregnancy. The WHO currently recommends screening for anaemia in pregnancy [14].

### Case coverage method

The screening method used for estimating vaccine efficacy has been applied recently as a surveillance tool for estimating drug effectiveness of SP-IPTp in reducing either low birthweight or anaemia prevalence during pregnancy [28]. The case-coverage method requires birthweight, anaemia, or placental malaria data and knowledge of frequency of use of SP-IPTp during pregnancy. It also has potential utility for estimating ITN effectiveness, providing data on ITN coverage in pregnant women is available and the frequency with which women use ITNs.

### Maternal mortality

This is an important complication of malaria in pregnancy and in lower transmission settings may result directly from severe malaria [29], or in higher transmission areas, indirectly, from severe anaemia [30]. With improved malaria control, maternal mortality indicators should improve and show reduced seasonal variation. As mortality indicators are routinely collected in many regions, there is a need for surveillance methodologies linking them to malaria intervention strategies and which allow seasonal analyses. Large sample sizes are needed for these analyses and population-based demographic health surveillance databases would be suitable.

### Operational and programmatic indicators

In order to improve access to antimalarial interventions targeting pregnant women, Reproductive Health Programmes are central to effective implementation. The high rate of antenatal attendance of at least two visits reported in sub-Saharan Africa is encouraging as an entry point for reaching pregnant women with antimalarial interventions. The gestational age of first visit and attendance of adolescents are two issues, which influence the effectiveness of programmes. This is because the burden of malaria in pregnancy is higher with late attendance as the intervention comes too late in pregnancy; also adolescents are the highest risk group due to both their young age and nulliparity. Adolescents living in malarious areas have poorer pregnancy outcomes compared to older women [31,32], and identification and evaluation of indicators in this vulnerable group is an important aspect of malaria control. There is also evidence in adolescent girls of a partial reversal during puberty of age-dependent malaria immunity (G. Kalanda, personal communication).

Although collection of antenatal attendance indicators is important, high attendance does not necessarily translate into high IPTp coverage [33] and updated information on SP-IPTp and ITN coverage and use among pregnant women living in high malaria transmission areas is essential to ensure that women in remote areas are accessing antimalarial interventions. Distance indicators should also be considered. There is evidence from rural Tanzania that mean birthweight for dispensary deliveries is associated with distance from the main district hospital, with deliveries occurring at 25 km or greater distances at highest risk for low birthweight [24]. ITPp was not implemented effectively in these rural districts and women further away from the main town may have received less health education regarding the consequences of malaria in pregnancy and had fewer opportunities for referral.

Strategies promoting SP-IPTp and ITN use for pregnant women are required. Indicators of health promotion activities through existing services should be assessed in areas of low coverage of interventions with a view to developing promotional activities targeted to pregnant women. There is evidence that these campaigns can lead to major increases in coverage [34], particularly as, even with knowledge of malaria in pregnancy, women also may express concerns that antimalarials have adverse effects on their baby and as a result can show reluctance to purchase these drugs [24]. Linked to this is the need for indicators of staff training, as well as research on the use of teaching aids, including diagrams which can be used as examples to show malaria burden estimates as illustrated in Figures 3 to 6.

### Monitoring safety of antimalarials

Monitoring the safety of antimalarial drugs during pregnancy is an essential part of public health programmes. WHO recommends the integration of pharmacovigilance in public health programmes to ensure their acceptance and effectiveness [35]. Evidence on the safety of drugs implemented by national malaria control programmes has the potential to increase programme success by providing guidance on reducing the risks of adverse reactions thereby increasing overall public trust. Furthermore, comparing the risk-benefit profiles of different antimalarial drugs would allow evidence-based decisions to be taken on the most effective as well as safer and better tolerated interventions. Adverse drug reactions, particularly serious adverse events affecting pregnancy outcomes (such as pregnancy loss or congenital anomalies) or severe skin reactions, can have serious public health impact and spoil confidence in any national programme. This can also lead to poor adherence, which in turn can result in treatment failure and increase the risk of development of parasite resistance leading to reduced efficacy.

Most countries in sub-Saharan Africa have little infrastructure and few resources for routine pharmacovigilance and very few have a formal system for routine collection of data on possible drug-related adverse effects [36,37]. The extrapolation of safety data derived from industrialised countries is limited by the type of drugs used in countries with established pharmacovigilance systems (i.e. with sparse experience of drugs for tropical diseases), the underlying differences in populations in terms of genetic make up, diet and health-seeking behaviour. Additionally, high levels of co-morbidities and concomitant medication use (such as for HIV, TB, malaria and other infections), as well as the use of traditional remedies in developing countries can increase the risk of drug interactions and adverse reactions. The concern is great for vulnerable groups like pregnant women. There is often limited information on the safety of drugs in this group as pregnant women are systematically excluded from pre-licensure clinical trials out of concern for not harming the mother or her unborn baby.

The process of establishing risk or safety of drugs used in pregnancy requires special considerations and involves the systematic recording of data on drug exposure and pregnancy outcomes. One of the most established approaches is the "Pregnancy Exposure Registry", a prospective, observational study monitoring outcomes of pregnancies exposed to specific drugs. This approach requires significant resources, but can be time-limited [38]. Much attention has been given to the establishment of an international antimalarial exposure registry due the widespread implementation of artemisinin combination therapies (ACTs) as first-line therapies in Africa and the uncertainty of their safety in early pregnancy [39]. There is a need to establish simple but effective pharmacovigilance systems to detect early signals of any major teratogenic effect associated with a prenatal exposure to antimalarial drugs. Such safety issues could have serious consequences for public health, and successful implementation of the recommended strategy for malaria in pregnancy requires reliable safety profiles for antimalarial drugs in use in order to inform policy makers, national malaria control programmes and health care providers. This will require close and effective collaboration between the different stakeholders (including policy makers, pharmacovigilance experts, the pharmaceutical industry and health care providers)[40]. Safety monitoring will assist in achieving the goals of national malaria control programmes and is thus an important indicator to be included in a monitoring and evaluation strategy.

### Research agenda

The indicators and assessment methodologies discussed in this review are summarized in Tables 2, 3, 4, which also include a summary of research priorities related to their evaluation. Although there is substantial evidence of the utility of some of these, there are few control programmes systematically using indicators for monitoring and evaluation of malaria control in pregnancy, largely due to a lack of investment, training and capacity. There are agreed research priorities to evaluate safe and effective drugs to treat malaria in pregnancy which include: identifying new drugs to replace sulphadoxine-pyrimethamine for IPTp; evaluating the optimum combinations of control measures in different epidemiological settings; ways for scaling-up use of ITNs and SP-IPTp [41]; and assessing the awareness of health workers on the risks of inadvertent treatments in the first trimester [42]. In parallel with these efforts, there is a need to introduce standardized monitoring and surveillance activities in pregnancy within national control programmes in order to ensure interventions remain effective when implemented on a programmatic basis.

**Table 2:** Monitoring and evaluation indicators, tools and assessment methods: output indicators.

**Indicator**	**Targets**	**Tools**	**Research**
**1. SP-IPTp coverage**			

Proportion of pregnant women with two SP-IPTp treatments in women delivering at health facilities or with home deliveries	80% of pregnant women receive IPTp in stable transmission areas	Health facility surveys and health promotion assessments	Missed opportunities; health promotion assessment; use of new promotion tools

			

**2. ITN coverage**			

Proportion of pregnant and non-pregnant women with ITNs, properly deploying (adherence) and properly treated with insecticides	100% of pregnant women at risk from malaria are protected	Health facility and community surveys; health promotion assessments	Missed opportunities; promotion assessment; use of new promotion tools

Proportion of adolescents deploying ITNs	100% coverage. New indicator	Female adolescent friendly health services (AFHS)	Evaluation of AFHS incorporating malaria control activities

Proportion using ITNs prior to first pregnancy	100% coverage. New indicator	Household surveys	Periodic cross-sectional surveys

			

**3. Proportion of pregnant women diagnosed and promptly treated with recommended drug**	All malaria patients correctly diagnosed and treated	Hospital surveys and case fatality rates	Adherence to first-line therapy

			

**4. Staff training**			

Proportion completing training programme	All ANC and malaria programme staff	Training programme	Curriculum content and feedback; use of diagrams illustrating malaria burden

**Table 3:** Monitoring and evaluation indicators, tools and assessment methods: impact indicators.

**1. Birthweight and anaemia**	**Targets**	**Tools**	**Research**
Low birthweight and maternal anaemia prevalence (Hb <10 g/dl and <8 g/dl) by parity	Reduced prevalence of low birthweight and anaemia	Rapid assessment methodologies; annual hospital wet season surveys	Birthweight and anaemia nomograms; annual trends and changes with new interventions

Malaria prevalence at delivery	Zero prevalence	Health facility surveys	Use of rapid diagnostic tests

			

**2. Maternal mortality**	Declining maternal mortality rates	Demographic health surveys and hospital case fatality rates	Seasonal mortality patterns and severe anaemia and malaria mortality rates

**Table 4:** Monitoring and evaluation indicators, tools and assessment methods: programmatic indicators.

**Indicator**	**Targets**	**Tools**	**Research**
**1. Identification of high risk individuals**			

Proportion of adolescent ANC attendees	>80% of adolescents attending ANC	Health facility assessments	Strategies for delaying pregnancies

Proportion of primigravidae with late ANC attendance	**<**10% attendees	Health facility assessment	Reasons for late attendance

Distance indicators of household residence from main health facility	Identification of high risk areas	Household surveys	Community SP-IPTp distribution

Antenatal HIV infection	Prevalence reduction	Mother to child HIV transmission assessments	Implementation of HIV prevention strategies

			

**2. Antimalarial drug efficacy estimates**			

Antimalarial therapeutic efficacy	Agreed surveillance interval (2–5 years)	Antimalarial *in vivo *sensitivity tests	Comparative drug evaluations

Proportional reduction in parasitaemia between first antenatal visit and delivery	New indicator	Health facility prevalence estimate	Comparison with birthweight or anaemia outcomes

Proportion of women with placental infection who have received two SP-IPTp doses	Assessment in sentinel health facilities	Case-coverage methodology for determining SP-ITPp efficacy	Comparison of case-coverage efficacy estimates method with *in vivo *tests

Antimalarial post-treatment prophylaxis	Agreed surveillance interval (2–5 years)	Antimalarial prophylactic efficacy tests	Evaluation in aparasitaemic women on IPTp

			

**3. Safety indicators**			

Inadvertent antimalarial drug exposure in first trimester	No exposures in asymptomatic women	Cross-sectional household/health facility surveys	Prevalence estimates and associated pregnancy outcomes

Assessment of safety indicators via national and international antimalarial exposure registry	Establishing functional registry	Standardized protocol under development by WHO	Demonstration sites; new methods for drug exposure, ascertainment, congenital anomaly detection

Whilst challenging, an effective partnership between the Ministries of Health and local research institutions is essential. The Ministry of Health could provide the necessary framework for routine health system data collection on the one hand (for output and outcome indicators), and research institutions and other partners providing the expertise for monitoring drug efficacy and programme effectiveness. Additional partners would be required to set up effective safety monitoring, such as policy makers and the pharmaceutical industry.

Tables 2, 3, 4 outline the tools available, and where and how each of the proposed indicators are collected, through health facilities, household surveys, sentinel surveillance or national monitoring. The frequency of collection of this information in part depends on the level of malaria exposure which women experience. This relates to their disease burden and levels of drug resistance, and also on the cost, level of complexity of the survey method, and whether the data would be collected through the health system or by separately conducted research studies. The translation of research findings into effective programmatic activities requires this on-going surveillance at these different levels. This should also ensure the equitable distribution of interventions to higher risk groups. Monitoring indicators should also be incorporated within relevant research studies in order to facilitate relating research study findings to concurrent programme evaluations.

Systematic monitoring should be co-ordinated at national and regional levels to facilitate summary analyses of basic indicators. Surveillance should encourage the formulation of appropriate databases, which could be updated with routinely collected pregnancy data. Data linkage would allow assessment of trends for seasonal indicators, allowing their utility to be assessed under changing epidemiological conditions. With increasing efforts towards the global eradication of malaria there is an urgent need for strengthening health management information systems for improved measurement and surveillance of control interventions in general, including special risk groups such as pregnant women and adolescents.

## Competing interests

The authors declare that they have no competing interests.

## Authors' contributions

BJB conceived the idea for the paper, prepared the outline for the analysis and the initial draft paper; MW prepared the draft for *in vivo *efficacy testing; UUW completed the literature search and prepared draft summaries; SD summarized the safety issues; JH reviewed the policy implications and assisted in drafting summary tables; SG provided original data from field studies in Burkina Faso and completed the required data analysis. All authors read and approved the final manuscript.
